# Effects of pyridoxine supplementation on severity, frequency and duration of migraine attacks in migraine patients with aura: A double-blind randomized clinical trial study in Iran

**Published:** 2015-04-04

**Authors:** Omid Sadeghi, Morteza Nasiri, Zahra Maghsoudi, Naseh Pahlavani, Masoud Rezaie, Gholamreza Askari

**Affiliations:** 1 Food Security Research Center AND Department of Community Nutrition, School of Nutrition and Food Science, Isfahan University of Medical Sciences, Isfahan, Iran; 2 Department of Research Committee, School of Nursing and Midwifery, Ahvaz Jundishapur University of Medical Sciences, Ahvaz, Iran; 3 Department of Research Committee, School of Nursing and Midwifery, Isfahan University of Medical Sciences, Isfahan, Iran

**Keywords:** Migraine with Aura, Pyridoxine, Headache, Iran

## Abstract

**Background: **Migraine is a chronic disease that affects nearly 6% of men and 18% of women worldwide. There are various drugs, which can successfully decrease migraine symptoms and frequency of migraine attacks, but these drugs usually are expensive. Hence, this study aimed to assess the effects of pyridoxine supplementation on severity, frequency and duration of migraine attacks as well as headache diary results (HDR).

**Methods:** This double-blind randomized clinical trial study was conducted on 66 patients with migraine with aura (MA) in Khorshid and Emam Mosa Sadr clinics of Isfahan University of Medical Sciences, Iran, in 2013. Patients were randomly allocated to receive either pyridoxine supplements (80 mg pyridoxine per day) or placebo. Severity, frequency and duration of migraine attacks and HDR were measured at baseline and at the end of the study.

**Results: **Mean age of patients was 34.24 ± 9.44 years old. Pyridoxine supplementation led to a significant decrease in headache severity (−2.20 ± 1.70 compared with −1 ± 1.50; P = 0.007), attacks duration (−8.30 ± 12.60 compared with −1.70 ± 9.60; P = 0.030) and HDR (−89.70 ± 134.60 compared with −6.10 ± 155.50; P = 0.040) compared with placebo, but was not effective on the frequency of migraine attacks (−2.30 ± 4 compared with −1.20 ± 7.80; P = 0.510).

**Conclusion: **Pyridoxine supplementation in patients with MA was effective on headache severity, attacks duration and HDR, but did not affect the frequency of migraine attacks.

## Introduction

Migraine is a debilitating and chronic disorder that affects 10-20% of the general population worldwide.^1^^,^^2^ This disorder is characterized by severe recurrent headache, nausea, vomiting, sensitivity to light and sound, neck pain and muscle tension.^3^^-^^5^ Migraine headaches are one-sided and throbbing that usually last between 4 and 72 h.^3^^,^^5^ This disease is more prevalent in women than men and often occurs in middle-aged individuals.^6^^,^^7^ Based on International Headache Society (IHS) criteria; there are two major classes of migraine: migraine with aura (MA) and migraine without aura.^3^ These two subtypes have the same symptoms, but 25% of patients with migraine perceive an aura, which is a transient disturbance in visual, sensory, language, or motor function and it can be defined as a signal of headache occurrence.^3^^,^^8^

Pathophysiology of migraine is influenced by genetic factors and environmental triggers.^9^^-^^11^ Lowering cerebral blood flow in the brain can induce depolarization waves in MA attacks. These waves spread across the brain cortex and stimulate trigeminovascular system (TVS). Activation of TVS can initiate attacks of head pain.^12^^,^^13^ Furthermore, vascular diseases, such as ischemic stroke, are considered as risk factors for MA.^14^^-^^16^ One of other factor that recent studies have shown its important in etiology of migraine is nutritional factors.^17^ It has been shown that high levels of serum homocysteine, obesity and nutritional intakes including caffeine, chocolate and tyramine as well as starvation can increase the incidence of MA.^17^^,^^18^ In addition, it’s suggested that pyridoxine, folate and cobalamin supplementations can play a role in severity and frequency of migraine attacks.^19^^,^^20^

Pyridoxine is a kind of vitamin B that is involved in several metabolism reactions. Previous studies have shown that pyridoxine administration improve vascular functions that are link to migraine attacks.^21^^,^^22^ However, there is no studies that assess the effects of pyridoxine supplementation on migraine profiles directly. Earlier studies have mostly focused on pyridoxine, folate and cobalamin combination.^19^^,^^20^ Furthermore; findings of these studies are inconsistent. One study reported that vitamin supplementation can decrease the severity and frequency of migraine attacks,^19^ while others reported no impact of vitamin intake on frequency of migraine attacks.^20^ Hence, due to scarce studies and conflicting results in this regard, the present study aimed to assess the effects of pyridoxine supplementation on characteristics of migraine attacks including severity, frequency, duration and headache diary results (HDR) in patients with MA.

## Materials and Methods

This double-blind randomized clinical trial study (IRCT2013060411763N9) was conducted on 66 patients with MA (12 men and 54 women) in Khorshid and Emam Mosa Sadr clinics of Isfahan University of Medical Sciences, Iran, from February 1^st^ through April 22^th^, 2013.

Age of 30-65 years old, Having history of migraine for a long time (˃ 5 years), 1 year history of severe, recurrent and long-lasting migraine attacks (at least one attack per month lasting 4 h) and current diagnosis of MA according to IHS criteria.^3^ Hence that studies have shown that homocysteine level in MA patients is positively associated with severity and frequency of migraine, and combine intake of pyridoxine, folate and cobalamin can reduce the mentioned symptoms in MA patients,^19^^,^^20^ we perform the study only on migraine patients who had aura. In this study, every patient having visual disturbance before head pain was considered as MA.

Current taking vitamin supplementation, clinical cardiovascular diseases, pregnancy occurrence and changing the supplement intake during the study.

Because of the lack of a similar study as a model, we estimated sample size based on the number of parameters of the regression model as 66 subjects. The study power was considered to be 80%.

Detailed information about age, medical history, taking medications and supplements were collected with a researcher-made checklist. Height was measured in a standing position without shoes by a tape measure with the nearest 0.5 cm. Weight was determined with minimal clothing and without shoes by analog scale with a precision of 100 g. Body mass index (BMI) was calculated as weight in kilograms divided by height in square meters. Waist circumference (WC) was measured by inelastic tape in the middle of bottom ribs and pelvic bones after a normal exhale.

Characteristics of migraine attacks including severity, frequency, duration (hour) and HDR were determined at baseline and at the end of the study. To measure the severity of MA attacks, we used visual analogue scale ranking the severity of a headache attack between 1 and 10.^23^ Number of migraine attacks in a month was considered as the frequency of attacks. To determine HDR index, we used the formula of frequency of attacks × duration of headache.^23^

Dietary intakes were assessed by means of 3 days food record at the 1^st^, 2^nd^ and the last week of study based on estimated values in household measurements. Nutrient intakes of participants were obtained using Nutritionist IV software (First Databank) modified for Iranian foods.

The incidence of adverse events was evaluated by recording all observed or volunteered adverse events. For this purpose, any study related adverse events during treatment were monitored by daily evaluation. For patients who withdrew or patients lost to follow-up, adverse events were acquired by telephone.

To carry out this study, after taking approve by Ethics Committee of Isfahan University of Medical Sciences, researcher referred to the clinics and selected patients, who had inclusion criteria, and after obtaining an informed consent from all the participants and providing verbal explanation about the research and assurance of confidentiality and anonymity, patients were randomly assigned to consume pyridoxine supplement (n = 33) or placebo (n = 33) for 12 weeks using envelopes containing numbers from a table of random numbers. Match of patients was done for age, gender, WC, BMI and characteristics of migraine attacks. Patients and investigators were not aware of allocated groups. Patients in pyridoxine group should consume 2 capsules containing 40 mg pyridoxine 2 times/day (80 mg pyridoxine/day) and patients in placebo group should consume 2 capsules of placebo containing lactose 2 times/day. Placebo capsules were similar in shape, color, and taste to pyridoxine capsule, which was produced in the School of Pharmacy, Isfahan University of Medical Sciences. We gave supplements to the participants in two stages (at the first and 6^th^ weeks) by someone except the researcher in the recruitment centers. All patients received pyridoxine supplementation or placebo in addition to routine treatment of migraine (pain killers or other anti-migraine drugs). Subjects’ compliance was measured through the remaining capsules at the end of the study using the following formula as: number of used capsules/ all given capsules × 100.

All statistical analyses were done by means of SPSS software (version 18, SPSS, Inc. Chicago, IL, USA). We applied Kolmogrov–Smirnov test to ensure the normal distribution of variables. To determine the differences in general characteristics and dietary intakes between pyridoxine and placebo groups, we used Independent-samples t-test. We used paired-samples t-test to determine the effects of pyridoxine and placebo on characteristics of migraine attacks including severity, frequency, duration and HDR. We applied Independent-samples t-test to compare the changes between pyridoxine and placebo groups. To assess the effects of age, gender and BMI on variables changes, we adjusted confounding variables by using analysis of covariance. P ˂ 0.050 was considered as a significant level.

## Results

***Follow-up***

**Figure 1:**
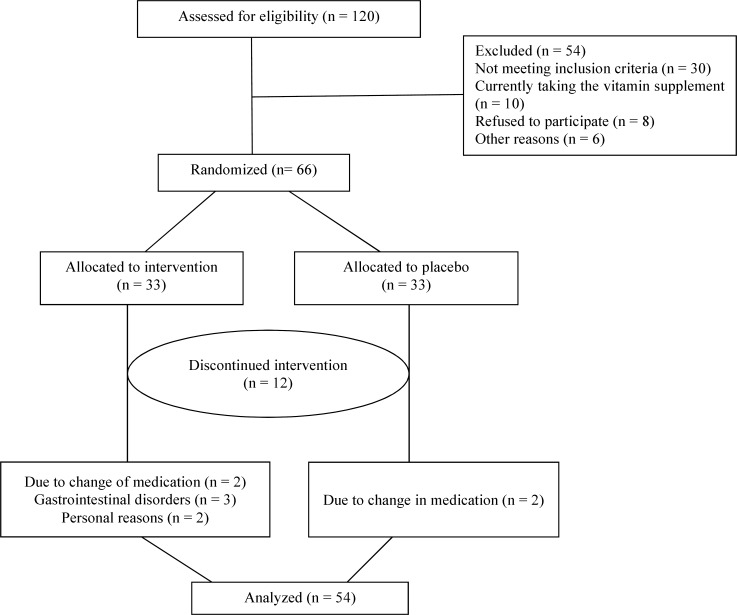
Flowchart showing allocation and exclusion of patients

In total, 10 patients in pyridoxine group were excluded due to change of medications (changing the routine treatment of migraine or vitamin supplementation) (n = 5), gastrointestinal disorders (heartburn) (n = 3) and personal reason (n = 2). Because of the change of medications (changing the routine treatment of migraine or vitamin supplementation), two patients were excluded from the placebo group. A total of 54 patients (23 in the pyridoxine group and 31 in the placebo group) completed the study, and they were considered for final analysis (Figure 1).

***Primary outcomes***

Mean of patients’ age, BMI and WC was almost 34.24 ± 9.44 years, 25.28 ± 4.28 kg/m^2^ and 82.80 ± 9.17, respectively. No significant difference was found in terms of age, BMI and WC between pyridoxine and placebo groups at the beginning of the study. In addition, baseline characteristics of migraine attacks including severity, frequency, duration and HDR between two groups were not different, significantly (Table 1). On the basis of 3 day's food record, mean dietary intakes were not different between those receiving pyridoxine and those receiving placebo (Table 2).

***Secondary outcomes***

Severity of migraine attacks decreased in both pyridoxine and placebo groups, significantly, but the reduction in the pyridoxine group was significantly more than a placebo group (-2.20 ± 1.70 compared with -1 ± 1.50; P = 0.007). Pyridoxine supplementation reduced frequency of migraine attacks, but as compared with a placebo group, this reduction was not significant (-2.30 ± 4 compared with -1.20 ± 7.80; P = 0.510). Intake of pyridoxine supplements led to a significant decrease in attacks duration (-8.30 ± 12.60 compared with -1.70 ± 9.60; P = 0.030) and HDR (-89.70 ± 134.60 compared with -6.10 ± 155.50; P = 0.040) compared with placebo group (Table 3). 

**Table 1:** General characteristics of patients with migraine with aura (MA) who received either pyridoxine supplement or placebo*

**Variables**	**Pyridoxine group** **	**Placebo group** ***	**P** £
Age (year)	35.39 ± 9.50	33.38 ± 9.45	0.440
BMI (kg/m^2^)	25.00 ± 3.85	26.24 ± 4.89	0.350
WC (cm)	80.55 ± 6.84	84.70 ± 10.51	0.110
Severity€	7.30 ± 0.87	7.00 ± 0.89	0.210
Frequency (per month)	10.30 ± 8.63	13.16 ± 12.02	0.310
Duration (h)	23.56 ± 16.86	17.35 ± 16.35	0.170
HDR§	222.60 ± 227.50	175.30 ± 167.40	0.380
Female (%)	19 (82.6)	24 (77.4)	0.450

BMI: Body mass index; WC: Waist circumference; HDR: Headache diary result;

*All values are expressed as mean ± standard deviation (SD) and number (percent);

** Received 80 mg vitamin B_6_ (2 capsules containing 40 mg vitamin B_6_) per day for 12 weeks;

*** Received 2 capsules of placebo per day for 12 weeks;

£ Obtained from independent sample t-test;

€ Measured by visual analogue scale that ranked headache severity from 1 to 10;

§ Determined by formula: frequency × duration

**Table 2:** Dietary intake of migraine with aura (MA) patients who received either pyridoxine supplement or placebo*

**Nutrients**	**Pyridoxine group** **	**Placebo group** ***	**P** §
Energy (kcal/d)	1957.70 ± 515.80	2191.00 ± 316.20	0.100
Protein (g/d)	68.70 ± 9.80	69.30 ± 8.80	0.840
CHO (g/d)	340.70 ± 33.40	364.90 ± 55.30	0.080
Fat (g/d)	72.20 ± 17.10	61.40 ± 25.90	0.110
Thiamine (mg/d)	2.01 ± 0.20	2.10 ± 0.30	0.370
Riboflavin (mg/d)	1.50 ± 0.30	1.40 ± 0.30	0.830
Niacin (mg/d)	22.80 ± 3.05	23.30 ± 4.50	0.650
Pyridoxine (mg/d)	1.30 ± 0.30	1.30 ± 0.40	0.90
Folate (µg/d)	244.30 ± 88.80	248.50 ± 99.90	0.880
Cobalamin (µg/d)	2.70 ± 1.10	2.60 ± 0.90	0.740
Magnesium (mg/d)	245.20 ± 46.90	244.20 ± 115.80	0.970
Calcium (mg/d)	865.00 ± 257.06	845.40 ± 212.40	0.780
Potassium (mg/d)	2885.30 ± 723.40	2901.50 ± 878.30	0.940
Tryptophan (mg/d)	568.80 ± 136.50	502.30 ± 173.01	0.160
EPA (mg/d)	9.40 ± 8.20	6.40 ± 5.30	0.160
DHA (mg/d)	28.20 ± 27.10	24.50 ± 12.70	0.560

CHO: Carbohydrate; EPA: Eicosapentaenoic acid; DHA: Docosahexaenoic acid;

* All values are expressed as mean ± standard deviation (SD) and number (percent);

** Received 80 mg vitamin B6 (2 capsules containing 40 mg vitamin B6) per day for 12 weeks;

*** Received 2 capsules of placebo per day for 12 weeks;

§ Obtained from independent sample t-test

**Table 3:** Characteristics of migraine attacks including severity, frequency, duration and headache diary result (HDR) at baseline and week 12 of migraine with aura (MA) patients who received either pyridoxine supplement or placebo*

**Group**	**Migraine characteristics**			
	**Severity** **	**Frequency**	**Duration**	**HDR** ***
Pyridoxine group§				
Before	7.30 ± 0.80	10.30 ± 8.60	23.50 ± 16.80	222.60 ± 227.50
After	5.04 ± 1.90	7.90 ± 7.05	15.20 ± 8.50	132.80 ± 169.30
Change€	-2.20 ± 1.70	-2.30 ± 4	-8.30 ± 12.60	-89.70 ± 134.60
P£	˂ 0.001	0.009	0.004	0.004
Placebo group¥				
Before	7.00 ± 0.80	13.10 ± 12.02	17.30 ± 16.30	175.30 ± 167.40
After	6.00 ± 1.40	11.90 ± 10.70	15.60 ± 11.50	169.20 ± 188.70
Change	-1.00 ± 1.50	-1.20 ± 7.80	-1.70 ± 9.60	-6.10 ± 155.50
P£	0.001	0.390	0.320	0.820
Pᶴ	0.007	0.510	0.030	0.040

HDR: Headache diary result;

* All values are expressed as mean ± standard deviation (SD) and number (percent);

** Measured by visual analogue scale that ranked the headache severity from 1 to 10;

*** Determined by formula: frequency × duration;

§ Received 80 mg vitamin B6 (2 capsules containing 40 mg vitamin B6) per day for 12 weeks;

€ Changes in each group are obtained by subtracting the week 12 value from the baseline value of each variable;

£ Obtained from paired t-test;

¥ Received 2 capsules of placebo per day for 12 weeks;

ᶴ Obtained from independent sample t-test

**Table 4:** Adjusted changes of characteristics of migraine attacks in migraine with aura (MA) patients who received either pyridoxine supplement or placebo*

**Variables**	**Vitamin B6 group** **	**Placebo group** ***	**P** £
Severity€	-2.28 ± 0.39	-1.04 ± 0.34	0.011
Frequency	-2.41 ± 1.58	-1.10 ± 1.35	0.480
Duration	-6.82 ± 2.63	-0.18 ± 2.25	0.037
HDR§	-82.44 ± 35.36	4.99 ± 30.17	0.040

HDR: Headache diary result; ANCOVA: Analysis of covariance;

* All values are means ± standard error (SEs) adjusted for age, gender and body mass index;

** Received 80 mg vitamin B6 (2 capsules containing 40 mg vitamin B6) per day for 12 weeks;

*** Received 2 capsules of placebo per day for 12 weeks;

£ Obtained from ANCOVA;

€ Measured by visual analogue scale that ranked the headache severity from 1 to 10;

§ Determined by formula: frequency × duration

When the analyses were adjusted for age, gender and baseline BMI, no significant changes were observed in our findings (Table 4).

***Adverse effects***

In this study, no side-effects of vitamin intake were reported at the end of the trial except the heartburn in 3 participants who stop the consumption of vitamin and excluded from the trial.

## Discussion

In this study, pyridoxine supplementation in MA patients resulted in a decrease in headache severity, attacks duration and HDR compared with placebo intake, but did not affect the frequency of migraine attacks, significantly. To the best of our knowledge, this study is the first study to examine the effects of pyridoxine supplementation on migraine attacks profiles including severity, frequency, duration and HDR.

Migraine is a chronic disease that affects nearly 6% of men and 18% of women worldwide. Migraine headache results in a substantial reduction in quality of life and it lead to heavy costs for migraine patients.^24^ There are various drugs, which can successfully decrease migraine symptoms and frequency of migraine attacks, but these drugs are often expensive and have many side-effects, and they are not always an effective treatment.^25^^,^^26^ It has been shown that some non-pharmacologic therapies such as relaxation, training, butterbur, riboflavin, magnesium, and coenzyme Q10 supplementation are effective to improve migraine symptoms.^25^^-^^27^ These methods often have a low risk of serious side-effects and they are less expensive than pharmacologic therapies. One of other kinds of supplementation, which its effectiveness was proven in combination with other vitamins in symptoms of MA patients is pyridoxine;^19^^,^^20^ however, data on the effects of single vitamin B_6_ supplementation are scarce.

In this study, we observed that pyridoxine supplementation lead to a reduction on severity and duration of migraine attacks as well as HDR, and it has no effects on attacks frequency. Our finding are supported by recent randomized, double-blinded placebo-controlled clinical trial by Menon et al., who done a 6 months trial of daily pyridoxine (25 mg), folic acid (2 mg) and cobalamin (400 µg) on 206 female patients diagnosed with MA. In this trial, a significant decrease was reported in headache severity and high migraine disability, after taking a 6 months vitamin supplementation compared with placebo intake while frequency of migraine attacks did not reduce significantly.^20^ In another similar clinical trial conducted by Lea et al., intake of pyridoxine, folate and cobalamin decreased severity and frequency of migraine attacks in addition to migraine disability significantly.^19^ Villegas-Salas et al., conducted a randomized, triple-blinded controlled trial to evaluate the effects of taking 150 mg pyridoxine supplements on severity of headache for 30 days. They found a significant reduction of headache severity in the pyridoxine group compared with a placebo group while no evidence on the effects of pyridoxine or vitamin Bs supplementation on attacks duration and HDR was observed.^28^

The exact mechanism explaining the beneficial effects of pyridoxine intake on MA symptoms is not clear. Earlier studies have shown that a point mutation in methylenetetrahydrofolate reductase (MTHFR) gene is more prevalent in MA patients.^29^ This mutation (MTHFR C677T) results a 50% reduction in MTHFR activity, which can induce serum hyperhomocysteinemia.^30^ It has been shown that reduced level of homocysteine can diminish headache severity, attacks frequency and migraine disability, too.^19^ Therefore, pyridoxine supplementation may improve MA symptoms, through reduction of serum homocysteine concentration.

Some limitations of our study need to be taken account. First, we did not measure the plasma pyridoxine and homocysteine levels because of limited financial resources, so it was not possible to diagnosis the patients who had vitamin B_6_ deficiency and it was not clear that vitamin supplementation could reduce the homocysteine levels. Therefore, it was not clear that pyridoxine supplementation decreased the migraine symptoms by lowering the homocysteine levels or by other possible mechanism. Second, we could not examine the effects of pyridoxine supplementation on other MA symptoms including nausea, vomiting, sensitivity to light and sound and especially aura symptom. Third, because of small sample size of the participants, we were unable to examine the favorable effects of pyridoxine supplementation is genders, separately. Hence, additional studies are required to provide more insight into our aims. In addition, the appropriate dosage of pyridoxine supplementation in patients with MA cannot be inferred from this study, and further studies are required.

## Conclusion

Pyridoxine supplementation in patients with MA was effective on headache severity, attacks duration and HDR, but did not affect the frequency of migraine attacks.

## Notes:

**How to cite this article: **Sadeghi O, Nasiri M, Maghsoudi Z, Pahlavani N, Rezaie M, Askari Gh. Effects of pyridoxine supplementation on severity, frequency and duration of migraine attacks in migraine patients with aura: A double-blind randomized clinical trial study in Iran. Iran J Neurol 2015; 14(2): 74-80.
